# Similar Outcomes in Males and Females Undergoing Surgery for Infective Endocarditis

**DOI:** 10.3390/jcm13174984

**Published:** 2024-08-23

**Authors:** Dror B. Leviner, Itay Schultz, Tom Friedman, Avishai Leizarowitz, Katia Orvin, Edward Itelman, Gil Bolotin, Erez Sharoni

**Affiliations:** 1 Department of Cardiothoracic Surgery, Carmel Medical Center, Haifa 3436212, Israel; esharoni@clalit.org.il; 2 The Ruth & Baruch Rappaport Faculty of Medicine, Technion, Haifa 3525433, Israel; itayschultz@gmail.com (I.S.); tomalach@gmail.com (T.F.); leizarowitz@campus.technion.ac.il (A.L.); g_bolotin@rambam.health.gov.il (G.B.); 3 Department of Cardiac Surgery, Rambam Health Campus, Haifa 3109601, Israel; 4 Department of Cardiology, Rabin Medical Center, Belinson Campus, Petah-Tikva 4941492, Israel; katiaorvin@gmail.com (K.O.); edi.itelman@gmail.com (E.I.)

**Keywords:** infective endocarditis, aortic valve replacement, mitral valve replacement, gender

## Abstract

**Background**: Sex-based differences in mortality have been previously observed in patients with surgically treated infective endocarditis. We sought to evaluate the risk factors leading to this difference. **Methods**: A retrospective cohort from three centers in Israel comprising 376 surgically treated patients, comparing short- and long-term mortality rates and risk factors between female and male patients. **Results**: Compared to male patients, female patients had higher rates of hypertension (62% vs. 48%), higher rates of Gram-negative infections (20% vs. 11%), and more mitral valve replacement (55% vs. 42%). Diabetes and age were the most significant predictors for mortality and did not differ between female and male patients. In-hospital mortality rates did not differ between female and male patients (29% vs. 26%), and the difference in long-term mortality was not statistically significant (46% vs. 36% *p* = 0.088). **Conclusions**: No statistical difference was observed in short- and long-term mortality between female and male patients, most likely due to a lack of difference in the rates of important risk factors such as diabetes and age. Mortality rates decreased in the last 10 years, and a good prognosis is observed for patients surviving the initial 30 days after surgery.

## 1. Introduction

Infective endocarditis (IE) is a serious and possibly deadly disease, with in-hospital mortality rates ranging around 10–20% and up to 40% for 1-year mortality rates [1,2,3]. Surgical valve replacement or repair (when feasible) is the treatment of choice for some of the patients’ IE [1,4]. According to the 2023 ESC guidelines for the management of IE, surgery is indicated mainly for patients with signs of heart failure, uncontrolled infection, and with a high risk of embolism or established embolism [5].

The notion that female patients suffer from the worst outcomes after surgery for IE in terms of in-hospital and short-term all-cause mortality has been studied before but has yielded contradicting results. While Bansal et al. [6] and Leterrier et al. [7] have reported that female sex is in itself a risk factor for mortality, Afshar et al. [8] have suggested that the higher comorbidity rates in female patients are the reason for this difference. This difference in outcomes was observed not only in patients who undergo surgery for IE but also in other thoracic surgery in general [9]. When addressing this difference, it is thought to be due to differences in the incidences of known comorbidities, treatment bias, and even in the microbial profile of the patients [8,10,11].

Since most studies in this matter are either based on single-center data, had a small number of patients, or originated in a US-based healthcare system, we have examined the difference in short- and long-term mortality between female and male patients in three independent hospitals in Israel. In particular, we sought to investigate whether the female gender is a risk factor for mortality after surgical treatment for IE.

## 2. Materials and Methods

Our cohort is a retrospective, multicenter registry of patients with IE. Inclusion criteria were patients who underwent surgical treatment between 31 December 2001 and 28 February 2023. Relevant data were extracted from the patient’s digital records and operative reports. Long-term follow-up was obtained by reviewing hospital medical records. Patients who were treated more than 4 weeks after their initial diagnosis were considered as having healed endocarditis and were excluded as well as patients with missing data on surgery and diagnosis. For patients with documentation of more than one case of IE, we included only the first episode. The patient’s diagnosis was established based on the modified Duke criteria. The patients’ age was recorded at the time of surgery for IE. Long-term mortality was defined as all-cause mortality occurring during the follow-up period. The follow-up time for survival was measured from the date of surgery to either the date of death or the date of the last contact with the patient. Our study was approved by the respective institutional ethics committee. The patients’ EF% was calculated using Simpson’s method.

The study was approved by the Carmel Medical Center, IRB number: 0172-21-CMC, approved on 6 June 2022. Informed consent for patients was waived due to the retrospective nature of the study.

### Statistical Analysis

Patients were grouped according to gender. Our primary outcome was all-cause mortality during initial hospitalization and up to 10 years of follow-up. Baseline characteristics of the patients that underwent surgical valve replacement were compared between male and female patients using the Wilcoxon rank sum test for continuous variables and Pearson’s χ^2^ test or Fisher’s exact test for categorical variables. A Kaplan–Meier (KM) model was used to plot the cumulative survivability for up to the median follow-up time. A logistic regression model was calculated to estimate the predictors for the in-hospital and long-term mortality and odds ratio (OR). The model included only statistically significant predictions and those which are relevant to the discussion. A Cox proportional hazards model with gender as the univariable model for hazard ratios (HRs) to in-hospital and long-term mortality was used. In order to assess the trends in mortality over time, patients were divided into 2 sub-groups: patients who were admitted between 2001 and 2013 (referred to as “Early period”) and patients who were admitted between 2014 and 2023 (comprising the last 10 years of cohort and referred as “Late period”). Finally, a multivariable model was calculated to address HRs for all confounders. A *p*-value < 0.05 was considered statistically significant. All of the analyses were performed using R (version 4.4.1) and R studio (version 2024.04.2+764).

## 3. Results

Our cohort included 1755 patients diagnosed with IE; 376 (21%) underwent surgical treatment. Of these, 262 (69%) were males with a mean age of 59 (±14), and 114 (31%) were females with a mean age of 60 (±15). Compared to male patients, female patients had a higher incidence of hypertension (62% vs. 48%, *p* = 0.011), beta-blocker use (56% vs. 41%, *p* = 0.021), and Gram-negative bacteremia (20% vs. 11%, *p* = 0.025), but a lower incidence of tobacco use (23% vs. 38%, *p* = 0.017), intravenous drug abuse (5.3% vs. 13%, *p* = 0.026), and Gram-positive bacteremia (55% vs. 68%, *p* = 0.023). Female patients had a slightly higher pre-operative ejection fraction (EF%) (61% ± 8 vs. 59% ± 9, *p* = 0.045) (Table 1).

A total of 202 patients underwent aortic valve replacement (AVR), of which 150 (74%) patients were male, and 157 patients had mitral valve replacement (MVR), of which 100 (63.7%) patients were male. Proportionally, female patients had lower rates of AVR and higher rates of MVR than male patients (50% vs. 63%, *p* = 0.031 and 55% vs. 42%, *p* = 0.025, respectively). Of patients who had MVR, female patients tended to have more biological prosthetic valves than male patients (82% vs. 63%, *p* = 0.016). In total, 20 patients had either double or triple valve surgery (9 females and 11 males) (Table 2).

**Figure 1:**
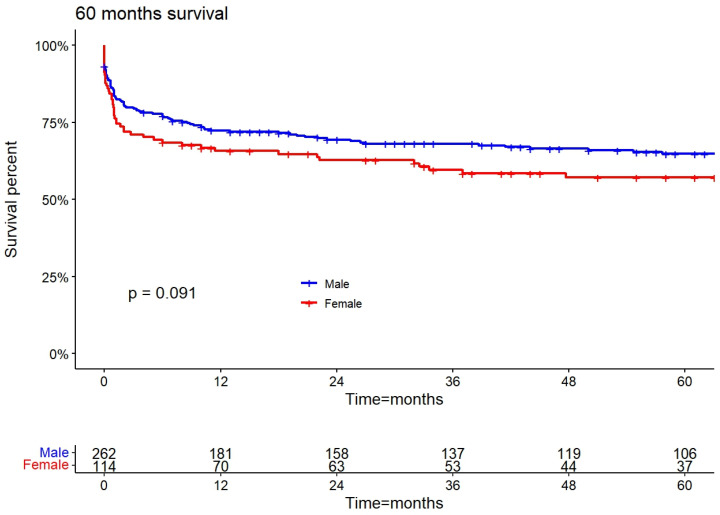
Kaplan–Meier plot for survivability after surgical valve replacement compared by gender.

The in-hospital mortality of the whole cohort was 27% (102 patients). Males and females had similar rates of in-hospital mortality at 26% and 29%, respectively. Female patients had higher rates of long-term mortality than male patients at 46% and 36%, respectively; however, this difference was not statistically significant (Table 3). The five-year KM survivability curves were lower but did not statistically differ between female and male patients (*p* = 0.091) (Figure 1).

Our logistic regression model showed that the female gender was not a statistically significant predictor for neither in-hospital nor long-term mortality. Diabetes, however, was a significant predictor for long-term mortality (OR 3.02, CI 1.33–7.07, *p* = 0.009), as well as intravenous drug use (IVDU) status (OR 7.24, CI 1.13–48.7, *p* = 0.037). Similarly, AVR was not a significant predictor of in-hospital mortality but was a risk factor for long-term mortality (OR 6.83, CI 1.97–26.2, *p* = 0.003) along with age (OR 1.04, CI 1.01–1.07, *p* = 0.026). Pre-operative moderate–severe tricuspid valve (TV) insufficiency was a good predictor for both in-hospital and long-term mortality (OR 4.58, CI 1.07–21.5, *p* = 0.045 and OR 6.16, CI 1.57–26.2, *p* = 0.011). Post-operative peak creatinine levels were associated with both in-hospital and long-term mortality (OR 1.84, CI 1.34–2.62, *p* < 0.001 and OR 1.70, CI 1.24–2.42, *p* = 0.002) (Table 4).

Our proportional hazard model showed that female gender was not a statistically significant factor for neither in-hospital nor long-term mortality (Supplemental Table S1).

Lastly, we have examined the difference in outcomes of our patients in the last 10 years and compared them with the patients in the time period between our earliest collection date up to the last 10 years. It showed a significant decrease in both in-hospital (42% vs. 22%, *p* < 0.001) and long-term mortality (58% vs. 33% *p* < 0.001) (Table 5). In light of this result, we have performed another Cox proportional hazard model that showed that patients in the last 10 years had significantly lower HR for both in-hospital and long-term mortality (Table 6).

## 4. Discussion

Our study was a three-center cohort that compared the characteristics and outcomes of male and female patients with IE after surgical valve replacement.

Female patients did not differ in the risk for short- and long-term mortality and female gender was not a predictor for short- and long-term mortality. The main predictors for short-term mortality were serum albumin levels and TV insufficiency. The main predictors for long-term mortality were diabetes, age, and post-operative maximal serum creatinine.

Female patients had lower rates of tobacco use, IVDU, Gram-positive bacteremia, and lower serum levels of creatinine, hemoglobin (HGB), and total bilirubin. Female patients also had higher rates of hypertension, beta-blocker use, Gram-negative bacteremia, and higher EF%. Female patients had lower rates of AVR and higher rates of MVR, with a higher percentage of biological valve use.

When exploring gender gaps in other cardiac disease processes, Xi et al. [12] found higher short-term mortality rates of females after ST-elevation myocardial infarction compared to males (relative risk 1.24) but not a significant difference in long-term mortality. They also found that the difference in risk factors such as diabetes, hypertension, hyperlipidemia, tobacco use, prior myocardial infarction, and prior percutaneous coronary intervention between sexes was not identified as a significant source of heterogeneity for short-term all-cause mortality. In coronary surgery, Harik et al. [13] performed a systemic review and found higher short-term mortality rates in women compared to men after coronary artery bypass grafting (CABG) (OR 1.26 with mortality of 4.8% vs. 2.7%, respectively). Those findings, which are compatible with the known literature, strengthen the notion that there are sex-based differences in morbidity and mortality in other cardiac diseases.

As opposed to findings in previous studies, our study did not show significant differences in all-cause short- and long-term mortality between female and male patients. Those works attribute this difference to surgery selection bias and initial clinical presentation including risk factors and the valve involved [14] (i.e., mitral valve vs. aortic valve vs. tricuspid valve). Sambola et al. [14,15] found that female patients were older than male patients (63 ± 16 vs. 58 ± 18), whereas our study shows no such difference (60 ± 15 vs. 59 ± 14). Chung et al. [9] also reported differences in risk factors such as age (women were older, had higher rates of hypertension and renal failure, and had higher ejection fractions). Our findings suggest that female patients did not differ in those risk factors that were found to be significant predictors for mortality, mostly diabetes and age, but also ejection fraction. Although there was a significant difference in the hypertension rates between females and males (62% vs. 48%), it was not found to be a risk factor for mortality after surgical valve replacement due to IE. Similarly, a systemic review of articles addressing sex differences in IE patients by Slouha et al. [11] on IE found that women were older (69.3 vs. 66) and had higher rates of diabetes and hypertension. Interestingly, they also found a higher incidence of IE in males compared to females (65.8% of IE patients were males, similar to what was observed in our study). Leterrier et al. [7] found a similar in-hospital mortality rate in females but a lower rate for males (23.2% vs. 17.3%), but when they performed multivariate analysis, they found age, antibiotic use prior to surgery, and staphylococcal IE but not female gender to be independent risk factors for hospital mortality. This report resembles our findings and also strengthens our notion that other comorbidities are the reason for the difference in short- and long-term outcomes for surgical treatment for IE. Our results suggest that aortic valve replacement is a risk factor for long-term mortality. We attribute this finding to the fact that AVR patients were slightly older and had higher post-operative plasma creatinine levels (Supplemental Table S2) which were by themselves independent risk factors for long-term mortality. It would be worth studying whether AVR is a major risk factor for mortality in IE patients or if it is a byproduct of the patient’s pre-operative risk status.

Our data show a significant decrease in mortality and HR for mortality in the last 10 years compared to the time period before that. When examining the known literature on the subject, the results are controversial; Toyoda et al. [16] have reported unchanged crude 90-day mortality rates and decreased adjusted 90-day mortality risks. On the other hand, Cresti et al. [17] reported a trend for increased mortality and related this trend to the aging population. Both works have discussed both surgically and medically treated patients with IE. We believe this reduced mortality is the result of better patient selection (mostly a more aggressive approach for earlier surgical intervention as highlighted in a recent guideline [5]), and improvements in intra-operative and post-operative management such as a more frequent use of fast-track extubation and a more aggressive use of CRRT to name just a few examples.

Another interesting point to address is the higher in-hospital mortality rate compared to the long-term mortality rate. As seen in Figure 1 and Table 3, most of the mortality was short-term (in-hospital and up to 30 days) and set around 27%, with no difference between male and female patients. In contrast, the cumulative long-term mortality rate was 39% (36–46%). Similar numbers were reported by Athela et al. [3] on both medically and surgically treated IE patients, suggesting that the long-term prognosis for IE patients is very good for those surviving the initial 30 days after diagnosis or surgery.

Our study has a few limitations. It is a retrospective cohort with all inherent limitations. The main limitation is the lack of assessment for selection bias since our inclusion criteria was surgical valve replacement and we do not have full access to all patients with IE who received only antibiotic treatment or those who were declined from surgery.

## 5. Conclusions

In this retrospective analysis from three cardiac surgery centers in Israel, we found no difference in short- and long-term mortality rates between males and females undergoing surgery for IE. We believe the main reason for this outcome is derived from the lack of differences in known risk factors in our study population. These findings may suggest that the underlying basis for the known difference in mortality results after any thoracic surgery and cardiac diseases is derived from differences in those risk factors. Decreased mortality rates were observed within the last 10 years of our cohort. Long-term prognosis is very high in patients who have survived 30 days after surgery. Further research is advised on the reason for the difference in the risk factors between sexes.

## Figures and Tables

**Table 1:** Patient characteristic.

Group	Characteristic	Female, N = 114 ^1^	Male, N = 262 ^1^	*p*-Value ^2^
Patient Characteristics	Age	60 (±15)	59 (±14)	0.2
	Over 65	55 (49%)	101 (39%)	0.093
	Diabetes	30 (26%)	71 (27%)	0.9
	Hypertension	71 (62%)	126 (48%)	0.011
	Dyslipidemia	31 (36%)	68 (35%)	0.9
	COPD	1 (1.2%)	11 (5.7%)	0.11
	CVA			>0.9
	Recent	11 (92%)	28 (90%)	
	Remote	1 (8.3%)	3 (9.7%)	
	Tobacco use	20 (23%)	73 (38%)	0.017
	Beta blocker	48 (56%)	78 (41%)	0.021
	IVDU	6 (5.3%)	34 (13%)	0.026
	Creatinine (mg/dL)	1.17 (1.14)	1.66 (1.65)	<0.001
	HGB (mg/dL)	9.94 (1.69)	10.71 (6.38)	0.015
	PLT (K/uL)	231 (111)	217 (121)	0.2
	Albumin (gr/dL)	2.99 (0.66)	2.96 (0.73)	>0.9
	Total bilirubin (mg/dL)	0.65 (0.57)	0.89 (0.99)	0.006
	CRP (mg/dL)	22 (41)	31 (54)	0.3
Culture Results	Culture-positive	87 (76%)	211 (81%)	0.4
	Gram-positive	63 (55%)	177 (68%)	0.023
	Gram-negative	23 (20%)	30 (11%)	0.025
Pre-op Echo Findings	EF, %	61 (±8)	59 (±9)	0.045
	AVI			0.2
	Moderate–severe	21 (47%)	77 (57%)	
	Trivial–mild	24 (53%)	57 (43%)	
	Aortic stenosis	22 (26%)	45 (24%)	0.7
	MVI			0.2
	Moderate–severe	39 (52%)	71 (43%)	
	Trivial–mild	36 (48%)	93 (57%)	
	MV stenosis	9 (11%)	10 (5.3%)	0.11
	TVI			0.021
	Moderate–severe	18 (27%)	20 (14%)	
	Trivial–mild	49 (73%)	125 (86%)	

^1^ Mean (SD); n (%); ^2^ Wilcoxon rank sum test; Pearson’s Chi-squared test; Fisher’s exact test. AVI = aortic valve insufficiency; COPD = chronic obstructive pulmonary disease; CRP = C-reactive protein; CVA = cerebrovascular accident; EF = ejection fraction; HGB = hemoglobin; IVDU = intravenous drug use; MV = mitral valve; PLT = platelets; MVI = mitral valve insufficiency; TVI = tricuspid valve insufficiency.

**Table 2:** Operative data.

Characteristic	Female, N = 114 ^1^	Male, N = 262 ^1^	*p*-Value ^2^
AVR	52 (50%)	150 (63%)	0.031
Valve type			0.4
Biological	42 (81%)	113 (75%)	
MVR	57 (55%)	100 (42%)	0.025
Valve type			0.016
Biological	47 (82%)	63 (64%)	
MVr	5 (4.8%)	12 (5.0%)	>0.9
TVR			0.2
Biological	9 (69%)	11 (48%)	

^1^ n (%); ^2^ Pearson’s Chi-squared test. AVR = aortic valve replacement; MVR = mitral valve replacement; MVr = mitral valve repair; TVR = tricuspid valve replacement.

**Table 3:** Mortality by gender.

Mortality	Female, N = 114 ^1^	Male, N = 262 ^1^	*p*-Value ^2^
In-hospital mortality	33 (29%)	69 (26%)	0.6
Long-term mortality	52 (46%)	95 (36%)	0.088

^1^ n (%); ^2^ Pearson’s Chi-squared test.

**Table 4:** Mortality by surgery period.

Mortality	Early Period, N = 103 ^1^	Late Period, N = 273 ^1^	*p*-Value ^2^
In-hospital mortality	43 (42%)	59 (22%)	<0.001
Long-term mortality	58 (56%)	89 (33%)	<0.001

^1^ n (%); ^2^ Pearson’s Chi-squared test.

**Table 5:** Logistic regression model of estimated predictors for in-hospital and long-term mortality.

	In-Hospital Mortality			Long-Term Mortality		
Characteristic	OR ^1^	95% CI ^1^	*p*-Value	OR ^1^	95% CI ^1^	*p*-Value
Female gender	1.39	0.55, 3.44	0.5	1.94	0.81, 4.66	0.14
Age	1.02	0.99, 1.05	0.2	1.04	1.01, 1.07	0.026
Diabetes	2.25	0.96, 5.38	0.064	3.01	1.33, 7.07	0.009
IVDU	1.56	0.22, 10.3	0.6	7.24	1.13, 48.7	0.037
Albumin	0.53	0.28, 0.96	0.044	0.43	0.23, 0.77	0.006
AV insufficiency						
Moderate–severe	0.54	0.16, 1.73	0.3	0.30	0.09, 0.93	0.039
TV insufficiency						
Moderate–severe	4.58	1.07, 21.5	0.045	6.16	1.57, 26.2	0.011
AVR	2.84	0.84, 10.1	0.10	6.83	1.97, 26.2	0.003
Post-op max creatinine	1.84	1.34, 2.62	<0.001	1.70	1.24, 2.42	0.002

^1^ OR = odds ratio, CI = Confidence Interval. AV = aortic valve; AVR = aortic valve replacement; IVDU = intravenous drug use; TV = tricuspid valve.

**Table 6:** Cox proportional hazard ratio for in-hospital and long-term mortality compared by surgery period.

Mortality	HR ^1^	95% CI ^1^	*p*-Value
In-hospital mortality	0.57	0.38, 0.85	0.006
Long-term mortality	0.64	0.46, 0.9	0.011

^1^ HR = hazard ratio, CI = Confidence Interval.

## Data Availability

The data related to this paper will be available upon request from the corresponding author.

## Supplementary Materials

The following supporting information can be downloaded at https://www.mdpi.com/article/10.3390/jcm13174984/s1, Table S1. Cox proportional Hazard’s ratio for In-hospital and long-term mortality compared by gender; Table S2. Age and post-operative max creatinine compared by surgery type.
