# Supplementary material for: Similar Outcomes in Males and Females Undergoing Surgery for Infective Endocarditis
Source: J Clin Med. 2024 Aug 23;13(17):4984. doi: 10.3390/jcm13174984 (PMC11396445; doi:10.3390/jcm13174984)
Supplement: Supplementary file 1 [file jcm-13-04984-s001.zip › jcm-3149135-supplementary.pdf]

Supplements

Supplemental Table S1. Cox proportional Hazard's ratio for In-hospital and long-term mortality compared by gender.

Mortality	HR[1]	95% CI[1]	p-value
In hospital mortality	1.15	0.76, 1.74	0.5
Long-term mortality	1.34	0.96, 1.88	0.090

[1]HR = Hazard Ratio, CI = Confidence Interval

Supplemental Table S2. Age and post-operative max creatinine compared by surgery type

Variable	AVR N = 182[1]	MVR N = 137[1]	p-value[2]
**Age**	61 ± 14	57 ± 15	0.017
**Post-Operative Max Cr**	1.63 ± 1.43	1.36 ± 0.91	0.080

[1]Mean ± SD

[2]Welch Two Sample t-test

AVR – Aortic valve replacement; Cr – Creatinine; MVR – Mitral valve replacement

Patients who had double or triple valve surgery were excluded from this calculation
